# Adipose derived stem cells and platelet rich plasma improve the tissue integration and angiogenesis of biodegradable scaffolds for soft tissue regeneration

**DOI:** 10.1007/s11033-020-05297-7

**Published:** 2020-02-18

**Authors:** N. Naderi, M. F. Griffin, A. Mosahebi, P. E. Butler, A. M. Seifalian

**Affiliations:** 1 UCL Centre for Nanotechnology and Regenerative Medicine, Division of Surgery & Interventional Science, University College London, London, UK; 2 Royal Free London NHS Foundation Trust Hospital, London, UK; 3 Director/Professor Nanotechnology & Regenerative Medicine, NanoRegMed Ltd, London, UK; 4 Plastic and Reconstructive Surgery Department, Royal Free Hospital, University College London, Pond Street, London, UK

**Keywords:** Soft tissue regeneration, Adipose stem cells, Platelet rich plasma, Biodegradable scaffolds, Nanocomposite scaffolds

## Abstract

Current surgical reconstruction for soft tissue replacement involves lipotransfer to restore soft tissue replacements but is limited by survival and longevity of the fat tissue. Alternative approaches to overcome these limitations include using biodegradable scaffolds with stem cells with growth factors to generate soft tissue. Adipose derived stem cells (ADSCs) offer great potential to differentiate into adipose, and can be delivered using biodegradable scaffolds. However, the optimal scaffold to maximise this approach is unknown. This study investigates the biocompatibility of nanocomposite scaffolds (POSS-PCL) to deliver ADSCs with and without the addition of growth factors using platelet rich plasma (PRP) in vivo. Rat ADSCs were isolated and then seeded on biodegradable scaffolds (POSS-PCL). In addition, donor rats were used to isolate PRP to modify the scaffolds. The implants were then subcutaneously implanted for 3-months to assess the effect of PRP and ADSC on POSS-PCL scaffolds biocompatibility. Histology after explanation was examined to assess tissue integration (H&E) and collagen production (Massons Trichome). Immunohistochemistry was used to assess angiogenesis (CD3, α-SMA), immune response (CD45, CD68) and adipose formation (PPAR-γ). At 3-months PRP-ADSC-POSS-PCL scaffolds demonstrated significantly increased tissue integration and angiogenesis compared to PRP, ADSC and unmodified scaffolds (p < 0.05). In addition, PRP-ADSC-POSS-PCL scaffolds showed similar levels of CD45 and CD68 staining compared to unmodified scaffolds. Furthermore, there was increased PPAR-γ staining demonstrated at 3-months with PRP-ADSC-POSS-PCL scaffolds (p < 0.05). POSS-PCL nanocomposite scaffolds provide an effective delivery system for ADSCs. PRP and ADSC work synergistically to enhance the biocompatibility of POSS-PCL scaffolds and provide a platform technology for soft tissue regeneration.

**Electronic supplementary material:**

The online version of this article (10.1007/s11033-020-05297-7) contains supplementary material, which is available to authorized users.

## Introduction

Soft tissue replacement is required in numerous clinical scenarios including breast and facial reconstruction, augmentation or correction of congenital deformities or following cancer resection [1]. It is widely expected that fat grafting, typically using the Coleman technique is the typical technique to apply fat grafts as a soft tissue filler [1]. This involves harvesting adipose tissue from the abdomen or thigh and then transferring it to fill the volume soft tissue defect. Using patient’s own adipose tissue for restoring soft tissue defects has multiple advantages including biocompatibility, feasibility, cost effectiveness and is abundantly available [1]. However, one drawback of fat grafting is the limited survival with time. Hence, the tissue engineering field has aimed to find alternative techniques to form adipose tissue for soft tissue replacement [1]. The implantation of a biodegradable scaffold with an appropriate stem cell, with or without the addition of growth factors is the most commonly investigated tissue engineering approach [1].

Identifying the optimal scaffold to deliver stem cells for adipose tissue engineering is unknown but several scaffolds have been evaluated including synthetic and biological scaffolds as well as decellularized scaffolds [2]. One highly tested synthetic polymer for adipose regeneration is Poly(e-caprolactone) (PCL), a biocompatible and biodegradable FDA approved polymer for medical devices [3]. Our group have modified PCL with polyhedral oligomeric silsesquioxane (POSS) nanoparticles to improve its hydrophobicity and inertness to form a nanocomposite polymer called POSS-PCL [4–7]. We have shown that POSS-PCL can support cell adhesion, proliferation and differentiation acting as an effective scaffold to support cell survival [4–7]. However, a successful biodegradable scaffold for adipose tissue engineering must integrate with the surrounding host tissue by integrating by laying down extracellular matrix and angiogenesis without causing an immune response or capsule formation to degrade effectively with time. The in vivo behaviour of POSS-PCL has not been fully explored to date [7].

Adipose derived stem cells (ADSC) are a source of adult stem cells within adipose tissue. ADSCs are of great research interest for the regenerative medicine field due to their ease of harvest, proliferation and differentiation potential to adipose tissue and their very favourable immunological properties [8]. An important property of ADSC in general is their capability to modulate the activation and proliferation of immune cells [9–14], which may have a significant impact on foreign body reactions to implanted synthetic scaffolds. Such properties of ADSCs have proven to be advantageous in the application of regenerative medicine [15]. We have previously demonstrated that POSS-PCL can support ADSC adhesion and proliferation. ADSCs have been widely implemented to differentiate into adipose tissue for regenerative applications.

Platelet-rich plasma (PRP) has been widely used across many clinical fields, especially for skincare and in aesthetic surgery. PRP contains several growth factors including epidermal growth factor, platelet derived growth factor (PDGF), transforming growth factor-β (TGF-β), vascular endothelial growth factor (VEGF), basic Fibroblast growth factor (bFGF), insulin-like growth factor, and keratinocyte growth factor. The high concentrations of these growth factors in PRP compared to that in normal plasma are responsible for the therapeutic effects of PRP [16, 17]. Many of these growth factors have important roles in the wound healing process and tissue regeneration. PRP stimulates the expression of type I collagen and matrix metalloproteinase-1 in dermal fibroblasts [18], and increases the expression of G1 cycle regulators, type I collagen, and matrix metalloproteinase-1 to accelerate wound healing [19]. Based on the above findings, this study considered whether ADSC transplantation in combination with PRP might improve the integration and vascularisation of the implanted POSS-PCL constructs in vivo and have the potential to form adipose tissue in situ.

Hence, this study aimed to evaluate the effectiveness of POSS-PCL as a scaffold to deliver ADSCs in vivo with and without a PRP coating. This study demonstrates that POSS-PCL scaffolds work in conjunction with PRP to improve the tissue integration and angiogenesis and form adipose when implanted subcutaneously in a rodent model over 3 months.

## Methods

### Fabrication of 3D POSS-PCL scaffolds

The POSS-PCL polymers were fabricated as 3D scaffolds using the phase separation/particulate-leaching technique as previously described (Supplementary Fig. 1) [6]. Sodium chloride (NaCl) was dissolved in 18% wt solution of POSS-PCL in DMAc containing Tween-20 surfactant. Stainless steel sieves were used to obtain a NaCl mixture of 150–250 µm. The final solution was then dispersed and degassed in a Thinky AER 250 mixer (Intertonics, Kidlington, UK). A 1:1 weight ratio of NaCl to polymer was used in all experiments. The polymer mixture was then spread evenly onto circular steel moulds. The sheets were submersed in deionised water to dissolve the solvent, initially 30 h. Following this period, frequent water changes were carried out to dissolve out the NaCl porogen particles and DMAc for 7 days. As a result, 8 cm × 8 cm circular polymer sheets with 5 mm thickness were synthesised. For experimental purposes the circular sheets of polymer were cut into 16 mm diameter disks to be used in 24-well plates, using a steel manual shape cutter.

### In vitro assessment

#### ADSC isolation and characterisation

For in vitro and in vivo experiments, allogenic rat ADSCs were isolated from the epididymal fat pads of 12-week old male Sprague-Dawley rats. ADSC were isolated according to the method described by Zuk et al. [20] with modifications. Following removal of fibrous tissue and visible blood vessels, 0.5 grams of epididymal fat samples were cut into small pieces (< 3 mm^3^) and digested in Dulbecco's Modified Eagle's Medium/Nutrient Mixture F-12 Ham (DMEM/F12) containing 300 U/ml crude collagenase I (Invitrogen, Life Technologies Ltd, Paisley, UK) for 30 min in an incubator (37 °C, 5% CO_2_). 10% Foetal Bovine Serum (FBS; Sigma, UK) was added to the dispersed material and filtered through 70 µm Cell Strainers (BD Biosciences, Oxford, UK). After centrifugation (290×*g*, 5 min), the ADSC-rich cell preparation formed a pellet at the bottom of the tube. The supernatant was removed and the pellet re-suspended. The rADSCs were then seeded at 13,000/cm^2^ for subculture.

ADSC from passage 0 were immunophenotypically characterised using flow cytometry as previously described [21]. ADSCs were then stained with antibodies for different CD (cluster of differentiation) antigens. Supplementary Table 1A lists the different antibodies, their fluorochrome, emission/excitation wavelength, clone, isotype, and dilution. 1 × 10^6^ cells at passage 0 per flow cytometry tube (6 separate tubes for each sample) were suspended in 0.2 ml Phosphate-buffered saline (PBS) and incubated with the antibodies for 30 min on ice and protected from light. At each analysis a separate tube was used for every antibody to provide compensation controls and one sample tube contained unstained cells as controls. Subsequently, EasyLyse solution was used to lyse the red blood cells − 3 ml of 1:20 Erythrocyte-Lysing Reagent EasyLyse (BD Biosciences, Oxford, UK) in distilled water was added to each tube, left in the dark at room temperature for 15 min, and then centrifuged at 515×*g* for 7 min at 5 °C. The supernatant was then removed, 3 ml of FACS buffer (PBS/0.2% BSA/0.05% sodium azide) added, and each tube was centrifuged at 515×*g*, 5 ºC for 7 min again. The supernatant was again removed and 200 µl of 1:3 diluted FACS fix solution (BD Biosciences, Oxford, UK) in distilled water was added. The samples were acquired using flow cytometry (MACSQuant® Analyzer 10; Miltenyi Biotec, Cologne, Germany) with machine settings as detailed in Supplementary Table 1B within 24 hours. Kaluza software (version 1.2; Beckman Coulter, USA) was used to analyse the data. Supplementary Fig. 2 shows that the cells were positive for CD90, CD44 and CD34 and negative for CD31 and CD45.

#### rADSC viability on scaffolds

The viability of seeded rADSCs on the various scaffolds was assessed using alamar blue viability assay (Life Technologies, UK) as previously described [21]. In brief, scaffolds were placed in 24 well plates and seeded with 25,000 ADSCs per scaffold. ADSC viability was then assessed on days, 2, 4, 7 and 14 days of in vitro culture using alamar blue dye according to the manufacturer’s instructions (n = 6). To control for background signal in the alamar blue assay, measurements from wells with medium only were evaluated.

#### rADSC proliferation on scaffolds

The proliferation of the seeded rADSCs was assessed using DNA assay on the various scaffolds as previously described [22]. In brief, scaffolds were placed in 24 well plates and seeded with 25,000 ADSCs per scaffolds. DNA content was then assessed after 1, 2, 4, 7 and 14 days of in vitro culture using the Fluoresecence Hoeschst DNA quantification kit (Sigma, UK) performed according to the manufacturer’s instructions (n = 6).

### In vivo assessment

#### Experimental design

POSS-PCL only samples without modification with PRP or ADSCs were included as a control group for this study. For the seeded samples in this experiment, ADSCs from 1 g of adipose tissue were expanded in proliferation medium (without PRP) on POSS-PCL scaffolds for 24 h prior to implantation. Immediately before implantation, the seeded scaffolds were rinsed in sterile PBS to remove residual proliferation medium. The top of the scaffolds were then seeded with ADSCs and referred to as ADSC. At 12 weeks post implantation, the rats were sacrificed by CO_2_ overdose and the scaffolds were explanted (n = 4/time point) within the surrounding tissues for histological analysis. Table 1 lists the different experimental groups included in this study.

**Table 1:** Experimental set up of the scaffolds used within the study.

Experimental groups				
Implanted constructs	POSS-PCL control	POSS-PCL & ADSC	POSS-PCL & PRP	POSS-PCL, ADSC & PRP
Time points (weeks)	12	12	12	12

#### Animals

All animals were treated with procedures approved by the local University College London (UCL) animal care committee under PL2020/7504 and experiments were conducted in accordance with the UK legislation on the protection of animals and the guidelines for the Care and Use of Laboratory Animals. For the implantation surgeries, male Sprague-Dawley rats were anesthetized with 2% isoflurane in 2 l/min of O_2_ and the incision site was marked with povidone-iodine. A 1 cm incision was made in the dorsal dermis of the rats and the scaffolds were carefully positioned in the subcutaneous space. The wounds were closed with 5/0 Monocryl dermal and subcuticular sutures. Each rat received 2 implants and both the seeded and unseeded scaffolds were assessed in quadruplicate (n = 6) at each time point. No adverse events were noted with any of the animals during anaesthesia or implantation. During the experiments, the animals were housed in groups and had free access to water and pellet food.

#### Preparation of platelet-rich plasma (PRP)

Platelet-rich plasma (PRP) preparation was performed as previously described with modifications [22, 23]. In brief, 20 ml of whole blood from allogenic 12-week old Sprague-Dawley rats was drawn percutaneously from the heart at the time of termination into tubes containing 3.8% sodium citrate. The tubes containing blood were centrifuged for 40 min at 200×*g*. The buffy coat, which contains PRP, in between the supernatant plasma and red blood cell layer, was collected into a neutral tube with a long pipette. PRP gelation was activated with a 10% calcium chloride solution and thrombin immediately before administration in vivo. The PRP was then coated on the top of scaffolds, allowing for 30 min incubation before implantation beneath the skin. An automated platelet counter showed that the platelet concentration in the PRP was 20.2 × 10^4^/ml, tenfold higher than the rat blood.

#### Assessment of renal and hepatic toxicity

To assess renal and hepatic toxicity 2-weekly blood samples were taken from the rats, which were implanted with POSS-PCL only controls to assess their renal and liver function throughout the study. Blood samples were obtained from the tails under aseptic techniques and examined for urea, creatinine, AST, ALT and ALP trends.

#### Haematoxylin and Eosin (H&E), Masson’s Trichrome (MT), CD31, alpha smooth muscle actin (α-SMA), CD45, and CD68 staining, Proliferator-activated receptor-γ (PPAR-γ)

POSS-PCL scaffolds within the surrounding skin and subcutaneous tissue were fixed in 4% paraformaldehyde for 24 h, rinsed with 70% ethanol, paraffin-embedded, and sectioned (8 ∝m sections). Representative sections were stained with H&E and Masson’s trichrome, according to standard procedures, to examine the collagen organization and scaffold integration into the host tissues, including analysis of cellular infiltration. In addition, scaffolds were stained against CD31 and α-SMA to demonstrate angiogenesis. The scaffolds were stained for CD68 for macrophages and CD45 for lymphocytes to examine the inflammatory response. Lastly, the scaffolds were stained against PPAR-γ for adipose staining at 12 weeks. Visualisation was performed using a Nanozoomer 2.0-RS Digital slide scanner C10730 (Hamamatsu, Japan). To quantify the extent of cellular integration into the scaffold, 3 fields of view (× 10 magnification) were chosen at random and the area of tissue stained by H&E and MT in the view was divided by the total field view to formulate the percentage of cellular integration and collagen deposition using Image J software (National Institute of Health, NIH). The same method was used to calculate the percentage of CD45^+^ and CD68^+^ cell infiltration in the scaffolds. To quantify the amount of inflammation around the implants, the thickness of the CD45^+^ and CD68^+^ cell layer in three fields of view per implanted scaffolds were measured using NDP View2 Nanozoomer Digital Pathology software (Hamamatsu, Japan). To quantify vessel formation, the methodology used was as per a previous study [24]. Briefly, the capillary number was calculated by identifying a positive endothelial cell cluster with a morphologically identifiable vessel with a lumen in three fields of view at × 10 magnification on each scaffold, providing 24 fields of view in total. Similarly, the number of positive PPAR-γ cells were identified in three fields of view at × 10 magnification on each scaffold, providing 24 fields of view in total to quantify the adipose staining at 12 weeks.

### Statistical analysis

All statistical analyses were performed using Prism software (Graphpad Software Inc., La Jolla, USA). Means and standard deviations were calculated from numerical data. In figures, bar graphs represent means, whereas error bars represent 1 standard deviation (SD). A *p* value of ≤ 0.05 was defined as the level of significance. The exact statistical analysis for each data set is described in the figure legend.

## Results

### In vitro assessment of rADSCs on POSS-PCL scaffolds

ADSCs showed significantly increased cell viability and DNA content on the POSS-PCL scaffolds with PRP compared to control scaffolds (p < 0.01) over a 14-day period (Supplementary Fig. 3).

### In vivo assessment of POSS-PCL scaffolds

Over 3 months there was no change in the animal’s health following implantation of the scaffolds. There was no signs of infection or extrusion of any of the implants over the 12 weeks.

### Collagen organisation and scaffold integration: H&E and MT staining

**Fig. 1:**
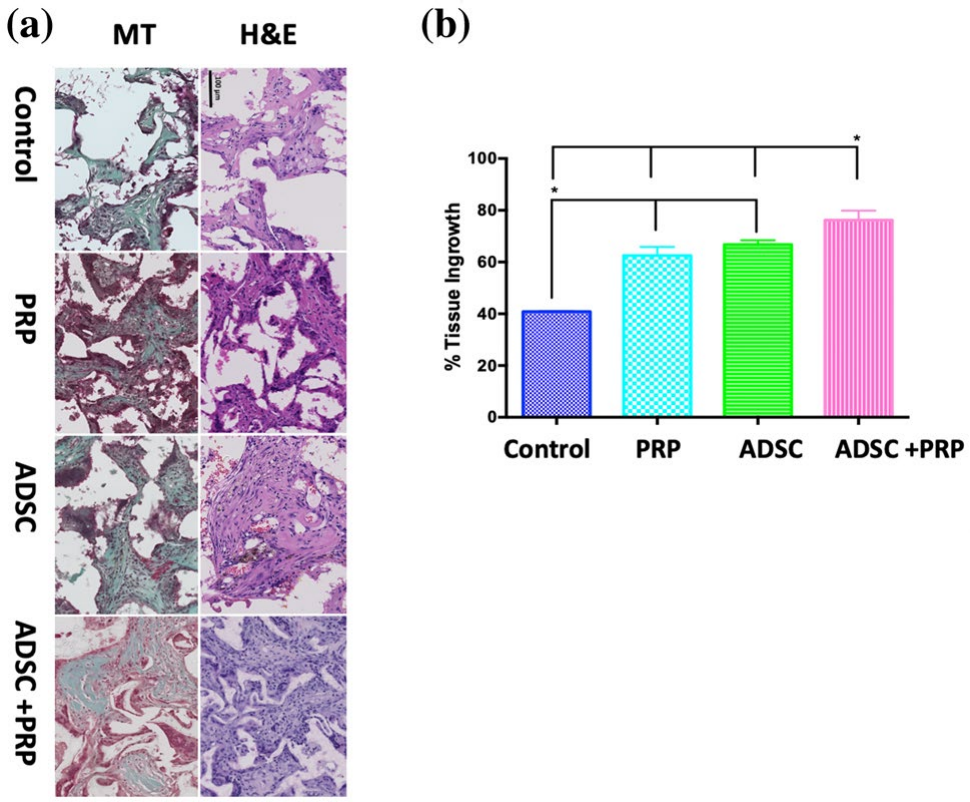
Tissue integration of the scaffolds assessed by H&E and collagen production assessed by Masson’s Trichome staining after 12 weeks of implantation. **a** Histological staining of the scaffolds at 12 weeks. **b** Quantification of cellular integration using H&E staining after 12 weeks of implantation. Note, the increase in tissue integration following implantation with PRP and ADSC compared to other scaffolds. (*p < 0.05)

In order to verify in vitro results and the effectiveness of POSS-PCL scaffolds to support ADSC proliferation, an in vivo study was undertaken for a period of 12 weeks in a rat model. Tissue ingrowth was confirmed using H&E staining, which showed after 12 weeks, ingrowth was significantly greater in in the POSS-PCL combined with ADSC and PRP experimental group compared to POSS-PCL with PRP, POSS-PCL with ADSC and control POSS-PCL groups (p < 0.05) (Fig. 1). In addition, control POSS-PCL scaffolds showed significantly less tissue integration & collagen production compared to all other groups (p < 0.05). Masson Trichrome confirmed greater positive collagen staining after 12 weeks for the same group (Fig. 1).

### Angiogenesis: CD31 and α-SMA

**Fig. 2:**
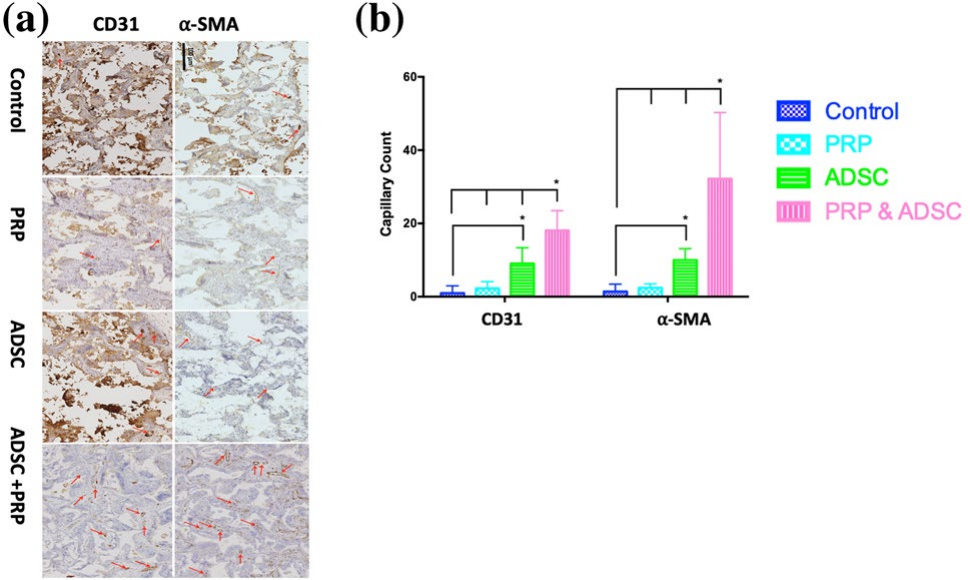
Vessel formation of the scaffolds assessed by CD31 and α-SMA after 12 weeks of implantation. **a** Histological staining of the scaffolds at 12 weeks. Brown-stained circular objects, representing capillaries are indicated with a red arrow. **b** Quantification of capillary formation using CD31 and α-SMA staining after 12 weeks. (*p < 0.05)

CD31 and α-SMA staining were used to demonstrate vascularisation of the scaffolds. At 12 weeks, vascularisation was significantly greater for scaffolds combined with ADSC and PRP compared to all other experimental groups (Fig. 2). In addition, scaffolds combined with ADSC had a statistically significant higher number of capillaries compared to control scaffolds (p < 0.05) (Fig. 2).

### Inflammation: CD45 and CD68

**Fig. 3:**
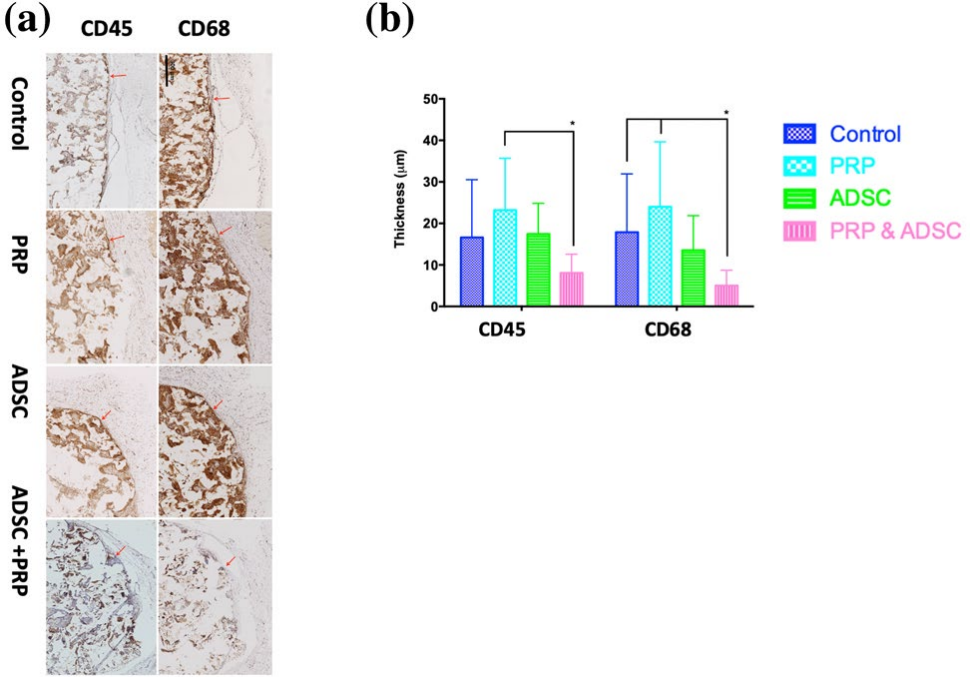
Implant-tissue interface and presence of inflammatory cells over the 12 weeks of implantation. **a** Histological staining for the CD68 and CD45 staining (brown) to demonstrate presence of inflammatory cells at the scaffold-tissue interface (red arrows). **b** Quantification of the CD45^+^ and CD68^+^ stained layer at the tissue-scaffold interface. PRP-ADSC-POSS-PCL group had a significantly thinner CD45^+^ stained layer at the interface compared to PRP-POSS-PCL group and a significantly thinner CD68^+^ stained layer compared to control and PRP-POSS-PCL (*p < 0.05)

**Fig. 4:**
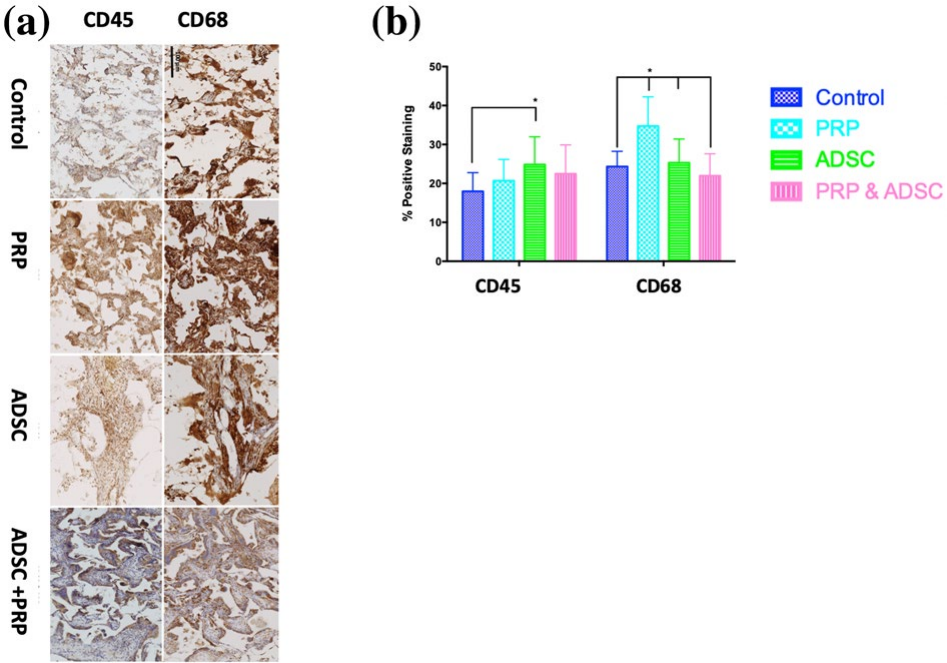
Presence of inflammatory cells within the implant over the 12 weeks of implantation. **a**Histological staining for the presence of CD45^+^ and CD68^+^ stained cells (brown) within the body of the scaffolds at 12 weeks. **b** Quantification of the CD45^+^ and CD68^+^ within the implant. ADSC-POSS-PCL group had significantly higher CD45^+^ staining compared to control POSS-PCL (p < 0.05), whilst PRP-POSS-PCL had significantly higher CD68^+^ staining compared to all other groups. (*p < 0.05)

CD45 and CD68 staining assessed inflammation and foreign body reaction around the implanted scaffolds. At 12 weeks, the implants were associated with minimal inflammation at the scaffold-tissue interface (Fig. 3). Quantification of the thickness of this layer showed that the PRP-ADSC-POSS-PCL group was associated with a significantly thinner CD45^+^ stained layer compared to PRP-POSS-PCL group and thinner CD68^+^ stained layer compared to PRP-POSS-PCL and control POSS-PCL groups (Fig. 3). Within the body of the polymer, the ADSC-POSS-PCL group had significantly higher CD45^+^ staining compared to control POSS-PCL (p < 0.05), whilst PRP-POSS-PCL had significantly higher CD68^+^ staining compared to all other groups (Fig. 4).

### Adipose Staining: PPAR-γ

**Fig. 5:**
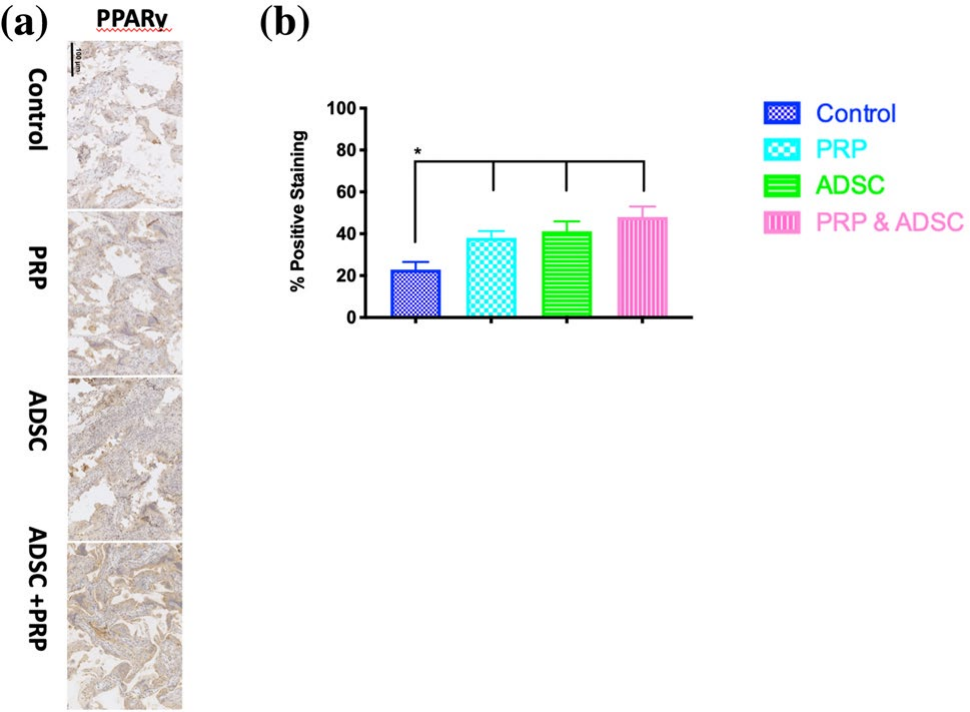
Adipose Staining of the scaffolds at 12-weeks of implantation. **a** Histological staining of the scaffolds for PPAR-γ over 12 weeks. **b** Quantification of the PPAR-γ. within the implant. PRP-ADSC-POSS-PCL had significantly greater adipose staining than all scaffolds at 12 weeks (*p < 0.01)

PPAR-γ was used to evaluate adipose staining at 12 weeks following implantation. It was observed that scaffolds with PRP + ADSC demonstrated the greatest staining, followed by ADSC alone and PRP, with the least staining showing on control scaffolds (p < 0.05) (Fig. 5).

### Renal and liver function monitoring

**Fig. 6:**
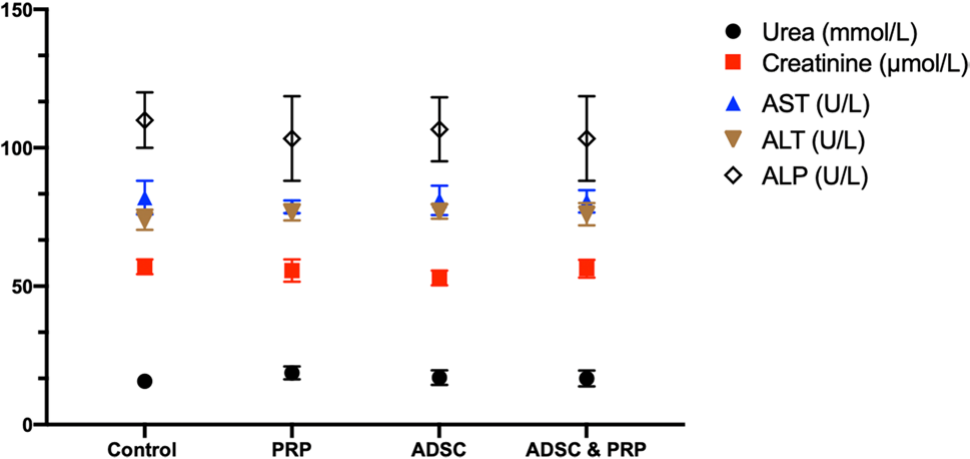
Renal and liver function values at 12 weeks of implantation. By 12 weeks there was no change in the liver and renal function between the scaffold types

As the scaffold was biodegradable, the liver and renal function was monitored over the 12 weeks (Fig. 6). Two-weekly blood samples taken to monitor changes in the renal and liver function values of the rats remained mostly stable throughout the study. There was an initially elevated ALP (162–184 U/L) at the start of the study which returned to normal by the next time point (2 weeks later) and remained steady throughout the rest of the study. In addition, AST was significantly elevated (128 U/L) in one of the rats 6 weeks post implantation. Also, AST returned to normal by the following time point.

## Discussion

In this study, we investigated the impact of ADSC seeding on POSS-PCL scaffold integration, angiogenesis, and host inflammatory response in an in vivo rodent model. Our results showed that the incorporation of ADSC with and without PRP enhances tissue ingrowth and integration of POSS-PCL scaffolds.

It was observed that ADSCs increase the angiogenesis of the biodegradable implants. Previous studies have reported similar results when using ADSC for tissue engineering purposes [25, 26]. Soft tissue regeneration is closely associated with the growth of vascular networks. An animal study investigating the angiogenic properties of cultured CD34+/CD13 + SVF cells from adipose tissue showed that these cells differentiated into endothelial cells, incorporated into vessels, and promoted both neovascularization and vessel-like structure formation in a Matrigel plug [27]. Similarly, nude mice with ischemic hindlimbs demonstrated marked perfusion improvement when treated with human ADSC (p < 0.05) [28]. This study also showed that ADSC secreted VEGF, hepatocyte growth factor and TGF-β. When ADSC were cultured in hypoxic conditions, VEGF secretion increased fivefold (p = 0.0016). The implantation of polymeric scaffolds without prior incorporation of a vascular network and blood flow through the scaffold similarly creates a hypoxic environment for ADSC. This may explain the encouraging results with regards to angiogenesis and tissue ingrowth in this study.

PRP did improve the tissue integration of the implants but not the angiogenesis of the implants alone. The in vitro assessment, demonstrated that scaffolds with PRP coating revealed an increase in cell viability and proliferation compared to scaffolds without PRP (p < 0.05). PRP scaffolds may have allowed greater cell adhesion and consequently greater proliferation in vivo. Other studies have also found that PRP increases ADSCs proliferation in an 3D environment [29, 30]. Although the use of PRP in combination with ADSC and POSS-PCL scaffolds corresponded to increased tissue ingrowth and vascularisation. Other studies have found that the PRP works synergistically with ADSCs in enhancing the tissue integration and vascularisation of implanted scaffolds [22]. We have previously examined the effect of PRP and ADSCs on non-biodegradable scaffolds, observing that such scaffolds tissue integration and angiogenesis is enhanced by ADSCs alone with PRP causing no effect [22]. Such differences may be accounted for the differences in the scaffold properties. The PRP may have been able to bind to the POSS-PCL in this study in optimal way to influence the in vivo environment. This highlights that the effect of surface modification tools can be varied among scaffold types. Furthermore, due to PRP composition varying between studies, it will be important to evaluate the PRP make-up in terms of growth factor type and concentration to understand the true efficacy of PRP. In addition, the immunodulatory properties of the PRP supplementation on ADSCs is important to evaluate.

When evaluating biodegradable scaffolds, the effect on the immune response of the host tissue is vitally important. The use of PRP without ADSC was associated with significantly higher infiltration of CD45^+^ and CD68^+^ inflammatory cells. PRP is defined as a preparation consisting of platelets concentrated in minimal volumes of plasma. PRP is used in various tissue-engineering procedures where it enhances regeneration. Growth factors from PRP have their source in alpha granules from platelets. In addition to growth factors [16, 18], a vast array of molecules including cytokines, chemokines, adhesive proteins, enzymes, and fibrinolytic and antifibrinolytic proteins is released from PRP. Platelet activation, an interaction between molecules such as collagen, thrombin, platelet-activating factor, serotonin, calcium, magnesium, thromboxane A2, and adenosine di-phosphate with platelet receptors, releases these biologically active molecules and growth factors. Upon activation, there is an initial ‘release’ burst that is later stabilised and maintained as a sustained discharge.

ADSC are known to have immunomodulatory properties [12, 31], including suppressing the proliferation of T lymphocytes [32], B lymphocytes, natural killer cells, and dendritic cells [33]. According to reported literature, the immunomodulatory properties of MSC may be enhanced when combined with PRP [34]. It has been shown that PRP used in MSC cultures delays the appearance of senescent phenotypes and protects from chromosomal instability [35–38]. It is plausible to favour the assumption that ADSC are stimulated by this gradual release of growth factors and biologically active molecules, but at the same time exert immunomodulatory effects inhibiting activation of inflammatory cells. Generally, the presence of inflammatory cells in scaffolds is closely related to the amount of tissue ingrowth. However, the combination of ADSC seeding and PRP addition to POSS-PCL scaffolds was associated with increased tissue ingrowth and angiogenesis and decreased inflammation and foreign body reaction.

Lastly, increased adipose tissue staining was observed at 12 weeks with PRP and ADSCs. The implants were implanted in the subcutaneous environment and thus the ADSCs may have either secreted required growth factors to generate adipose tissue or differentiated into adipose tissue to form soft tissue. To date, minimal evidence has demonstrated the in-situ capacity of ADSCs to form adipose tissue without differentiation medium as shown in this study [39]. Fang-Tian et al. demonstrated that ADSCs with PRP and ginsenoside Rg1 on collagen type I scaffolds could support adipocyte differentiation in vivo after 3 months of implantation in a nude mouse model [39]. However, further studies are needed to understand the potential of ADSCs to form adipose in vivo without the supporting signals of differentiation medium.

This study highlights the potential of POSS-PCL as a scaffold for soft tissue regeneration when utilised in combination with PRP [40]. To date, the ideal biomaterial design for adipose regeneration is unknown with various materials being investigated including natural bio-polymer hydrogels such as collagen, gelatin and glycosaminoglycans [40]. Such materials are advantageous as they allow for the incorporation of peptide sequences to improve adipose regeneration such as Arg-Gly-AsP (RGD) [40]. However, one drawback to such materials is the lack of control of mechanical properties compared to synthetic materials [40]. Studies have shown that when the mechanical properties of the scaffold are similar to adipose tissue the differentiation of the ADSCs improves [40]. Thus, POSS-PCL is a promising scaffold for soft tissue regeneration as it allows for tight control of mechanical properties and allows for the incorporation of growth factors to support adipose differentiation [40].

In conclusion this study shows that, PCL scaffolds demonstrated the ability to integrate into their surrounding tissue, support tissue ingrowth and angiogenesis, and induce minimal inflammation. Implantation of POSS-PCL scaffolds in combination with ADSC and PRP was associated with significantly higher tissue ingrowth and angiogenesis. This is mostly likely to be due to the release of biologically active molecules and growth factors from PRP as well as immunomodulatory effects of ADSC. In addition, there were no cases of extrusion or rejection of the implanted scaffolds and no systemic toxicity observed in any of the animals. POSS-PCL in combination with ADSC and PRP would provide a suitable platform for soft tissue regeneration.

## Electronic supplementary material

Below is the link to the electronic supplementary material.

Supplementary material 1 **Supplementary Fig. 1**. Scanning Electron Microscopy (SEM) imaging of the POSS-PCL scaffolds utilised in this study. Scale bar: 200 ∝m. (PNG 671.6 kb)

Supplementary material 2 **Supplementary Fig. 2**. Immunophenotype of rat ADSC population among SVF cells. These cells stained CD44^+^/CD34^+^/CD90^+^/CD45^−^/CD31^−^ (Figure reproduced from [22]). (PNG 481.2 kb)

Supplementary material 3 **Supplementary Fig. 3**. Cell viability [A] and proliferation [B] of the rat adipose derived stem cells in vitro culture over 14 days. Note the increase in cell viability and proliferation over 14 days. *p < 0.05 (PNG 160.1 kb)

Supplementary material 4 **Supplementary Table 1. Flow Cytometry Analysis used within the study. [A]**. Details of flow cytometry antibodies including their fluorescent dye, excitation wavelength, and dilution. FITC: fluorescein isothiocyanate, APC: allophycocyanin, PE: phycoerythrin. **[B]**. Flow cytometry voltage configurations. FSC: forward scatter, SSC: side scatter, FITC: fluorescein isothiocyanate, PE: phycoerythrin, APC: allophycocyanin (Table reproduced from [22]). (DOCX 13.6 kb).
