# Supplementary figures and images for: Adipose derived stem cells and platelet rich plasma improve the tissue integration and angiogenesis of biodegradable scaffolds for soft tissue regeneration
Source: Mol Biol Rep. 2020 Feb 18;47(3):2005–13. doi: 10.1007/s11033-020-05297-7 (PMC7688190; doi:10.1007/s11033-020-05297-7)

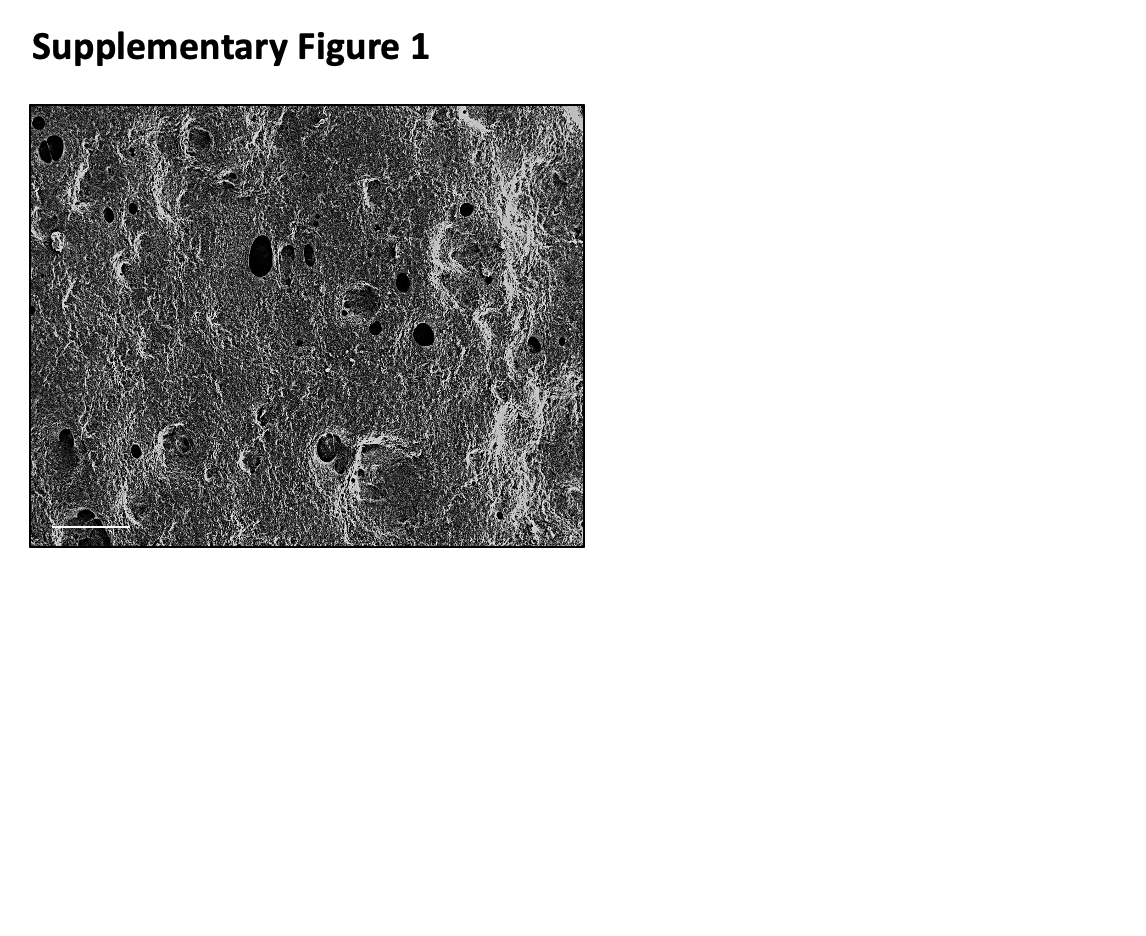

Supplement: Supplementary file 1 — Supplementary material 1 Supplementary Fig. 1. Scanning Electron Microscopy (SEM) imaging of the POSS-PCL scaffolds utilised in this study. Scale bar: 200 ∝m. (PNG 671.6 kb) [file 11033_2020_5297_MOESM1_ESM.png]

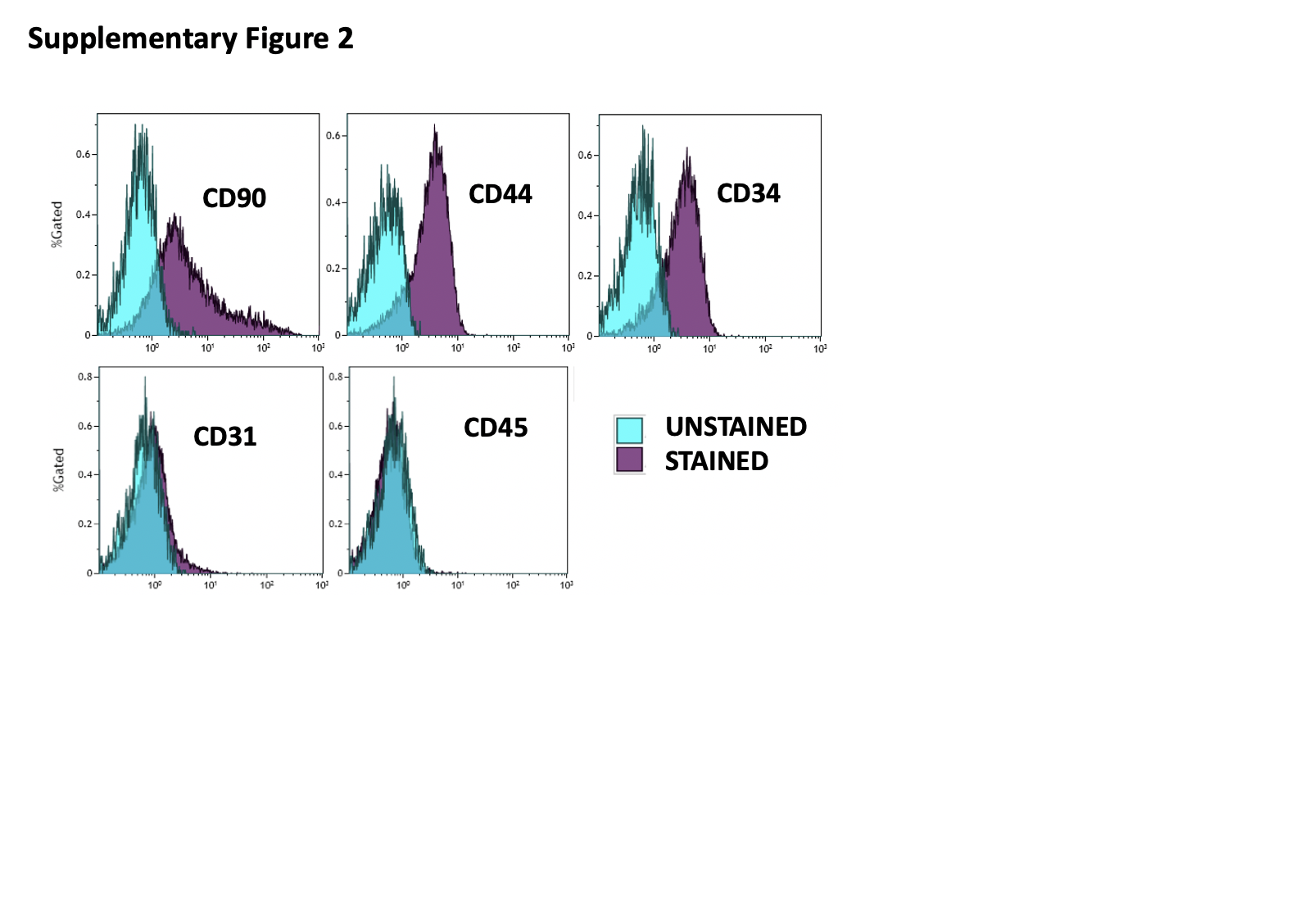

Supplement: Supplementary file 2 — Supplementary material 2 Supplementary Fig. 2. Immunophenotype of rat ADSC population among SVF cells. These cells stained CD44+/CD34+/CD90+/CD45−/CD31− (Figure reproduced from [22]). (PNG 481.2 kb) [file 11033_2020_5297_MOESM2_ESM.png]

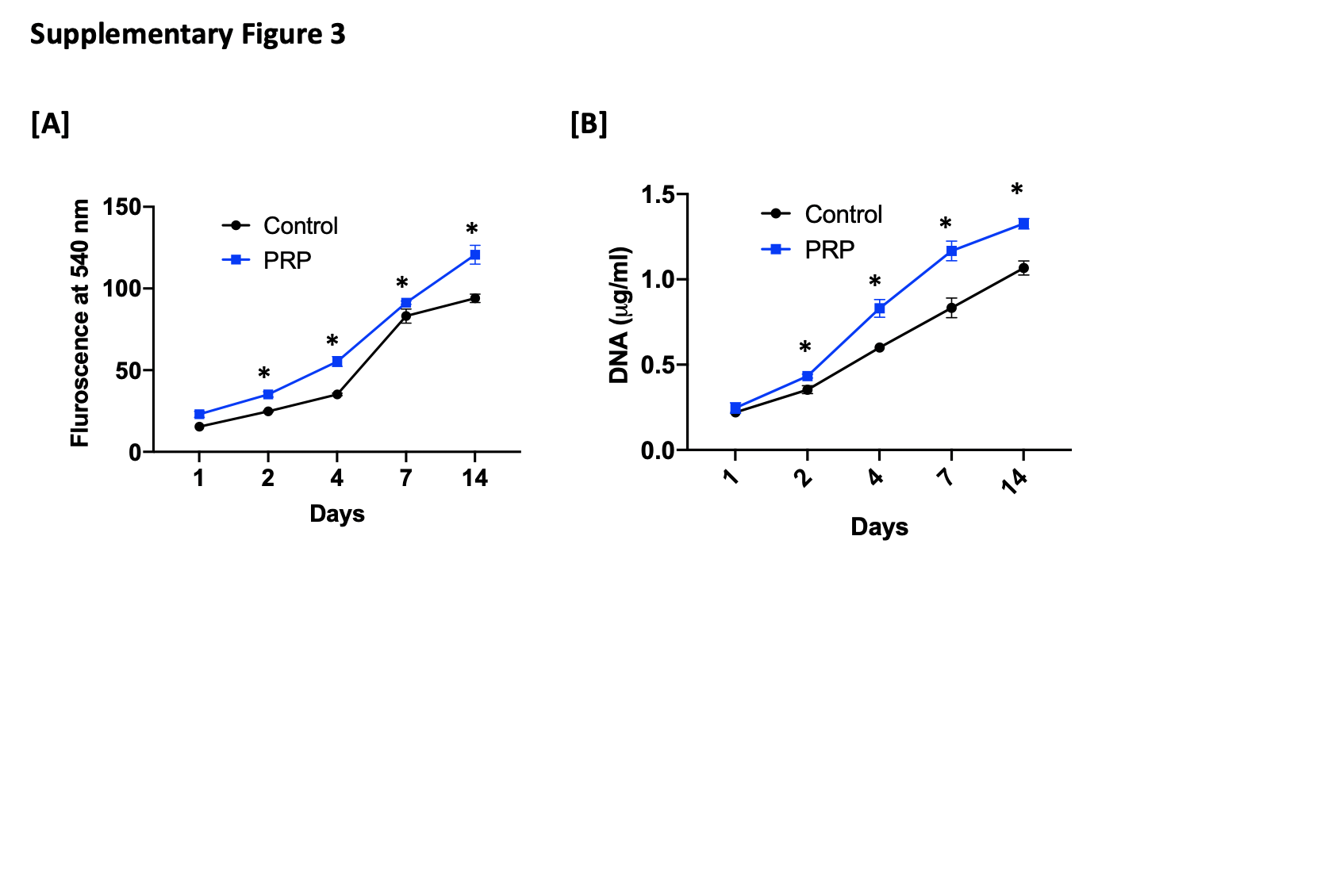

Supplement: Supplementary file 3 — Supplementary material 3 Supplementary Fig. 3. Cell viability [A] and proliferation [B] of the rat adipose derived stem cells in vitro culture over 14 days. Note the increase in cell viability and proliferation over 14 days. *p < 0.05 (PNG 160.1 kb) [file 11033_2020_5297_MOESM3_ESM.png]
