# Supplementary material for: Adipose derived stem cells and platelet rich plasma improve the tissue integration and angiogenesis of biodegradable scaffolds for soft tissue regeneration
Source: Mol Biol Rep. 2020 Feb 18;47(3):2005–13. doi: 10.1007/s11033-020-05297-7 (PMC7688190; doi:10.1007/s11033-020-05297-7)
Supplement: Supplementary file 4 — Supplementary material 4 Supplementary Table 1. Flow Cytometry Analysis used within the study. [A]. Details of flow cytometry antibodies including their fluorescent dye, excitation wavelength, and dilution. FITC: fluorescein isothiocyanate, APC: allophycocyanin, PE: phycoerythrin. [B]. Flow cytometry voltage configurations. FSC: forward scatter, SSC: side scatter, FITC: fluorescein isothiocyanate, PE: phycoerythrin, APC: allophycocyanin (Table reproduced from [22]). (DOCX 13.6 kb). [file 11033_2020_5297_MOESM4_ESM.docx]

**Supplementary Table 1**

|  | **CD90** | **CD44** | **CD34** | **CD31** | **CD45** |
| --- | --- | --- | --- | --- | --- |
| Source | Abcam, Cambridge, UK | | | | |
| Clone | HIS51 | OX-50 | ICO-115 | TLD-3A12 | MRC OX-1 |
| Isotype | IgG2a | IgG1 | IgG1 | IgG1 | IgG1 |
| Fluorochrome | APC | PE | PE | FITC | FITC |
| Concentration | 1:1 | 1:1 | 1:1 | 1:1 | 1:1 |
| Emission/ Excitation wavelength (nm) | 660/645 | 575/488 | 575/488 | 528/493 | 528/493 |

| **Flow Cytometry Configuration** | |
| --- | --- |
| Parameters | Voltage |
| FSC | 0 |
| SSC | 269 |
| FITC | 385 |
| PE | 440 |
| APC | 610 |
